# Autoimmune Pulmonary Alveolar Proteinosis in a Patient With Chronic Disseminated Blastomycosis: A Case Report

**DOI:** 10.7759/cureus.109042

**Published:** 2026-05-17

**Authors:** Michael Obregon, Samar Waqar, M. Haitham Bakir, Zainab Alnafoosi, Ali Zubairi

**Affiliations:** 1 Pulmonary and Critical Care Medicine, Southern Illinois University School of Medicine, Springfield, USA; 2 Infectious Diseases, Southern Illinois University School of Medicine, Springfield, USA; 3 Infectious Diseases, Southern Illinois University School of Medicine, Springfield , USA; 4 Pulmonary and Critical Care Medicine, Aga Khan University Hospital, Karachi, PAK

**Keywords:** autoimmune pulmonary alveolar proteinosis (pap), crazy-paving pattern, disseminated blastomycosis, disseminated fungal infection, granulocyte-macrophage colony-stimulating factor (gm-csf) autoantibody, pulmonary alveolar proteinosis (pap)

## Abstract

Pulmonary alveolar proteinosis (PAP) is a rare diffuse lung disease characterized by the intra-alveolar accumulation of surfactant, leading to progressive gas exchange impairment. While autoimmune PAP, driven by granulocyte-macrophage colony-stimulating factor (GM-CSF) autoantibodies, accounts for the majority of cases, its association with chronic fungal infections is exceedingly rare. Only one prior case of PAP occurring in the setting of active blastomycosis has been documented since 1995. We present a unique case of a patient with chronic disseminated blastomycosis who developed worsening respiratory failure due to the development of autoimmune PAP. It highlights the importance of broadening the differential diagnosis when patients with known pulmonary infections fail to respond to appropriate antimicrobial therapy.

## Introduction

Pulmonary alveolar proteinosis (PAP), first described by Rosen and Castleman in 1958 [1], is a rare, diffuse lung disease characterized by the accumulation of surfactant in the alveoli, leading to impaired gas exchange. Patients may present with nonspecific symptoms such as progressively worsening dyspnea and cough to outright respiratory collapse [2]. The prevalence is estimated at 6 to 7 cases per million in the United States [3]. PAP is driven by the dysfunction of alveolar macrophages, which are responsible for surfactant catabolism [4].

PAP is broadly classified into three categories: primary (autoimmune), secondary, and congenital [2]. Autoimmune PAP, which accounts for approximately 90% of cases, is caused by elevated levels of granulocyte-macrophage colony-stimulating factor (GM-CSF) autoantibodies that neutralize the signaling required for macrophage maturation [4]. Secondary PAP occurs in the setting of underlying conditions such as hematologic malignancies, immunodeficiency, or environmental exposures that functionally impair alveolar macrophages without the presence of autoantibodies [5]. Congenital PAP results from mutations affecting surfactant production and is mainly diagnosed in children [6].

Blastomyces dermatitidis is a dimorphic fungus endemic to the Ohio and Mississippi River valleys in the United States. Most infections are acquired via environmental exposure. Several species have been identified as causative agents of human disease: *Blastomyces gilchristii*, *Blastomyces helicus*, *Blastomyces percursus*, *Blastomyces emzantsi*, and *Blastomyces parvus*; however, *B. dermatitidis* remains the most clinically significant [7]. While pulmonary infection is common, the association between active blastomycosis and PAP has been reported in a single case report by Kellar et al. in 1995 [8]. We present a case of a patient with chronic disseminated blastomycosis who developed worsening respiratory failure and was subsequently diagnosed with autoimmune PAP. This article was previously presented as an oral presentation at the 2025 CHEST Annual Scientific Meeting on October 21, 2025.

## Case presentation

A 46-year-old male presented to the emergency department (ED) with a one-year history of dyspnea on exertion that had acutely progressed over the preceding two weeks. His past medical history was significant for disseminated blastomycosis diagnosed three years prior, with involvement of the skin, lungs, larynx, and central nervous system (CNS).

Prior history of blastomycosis

**Figure 1:**
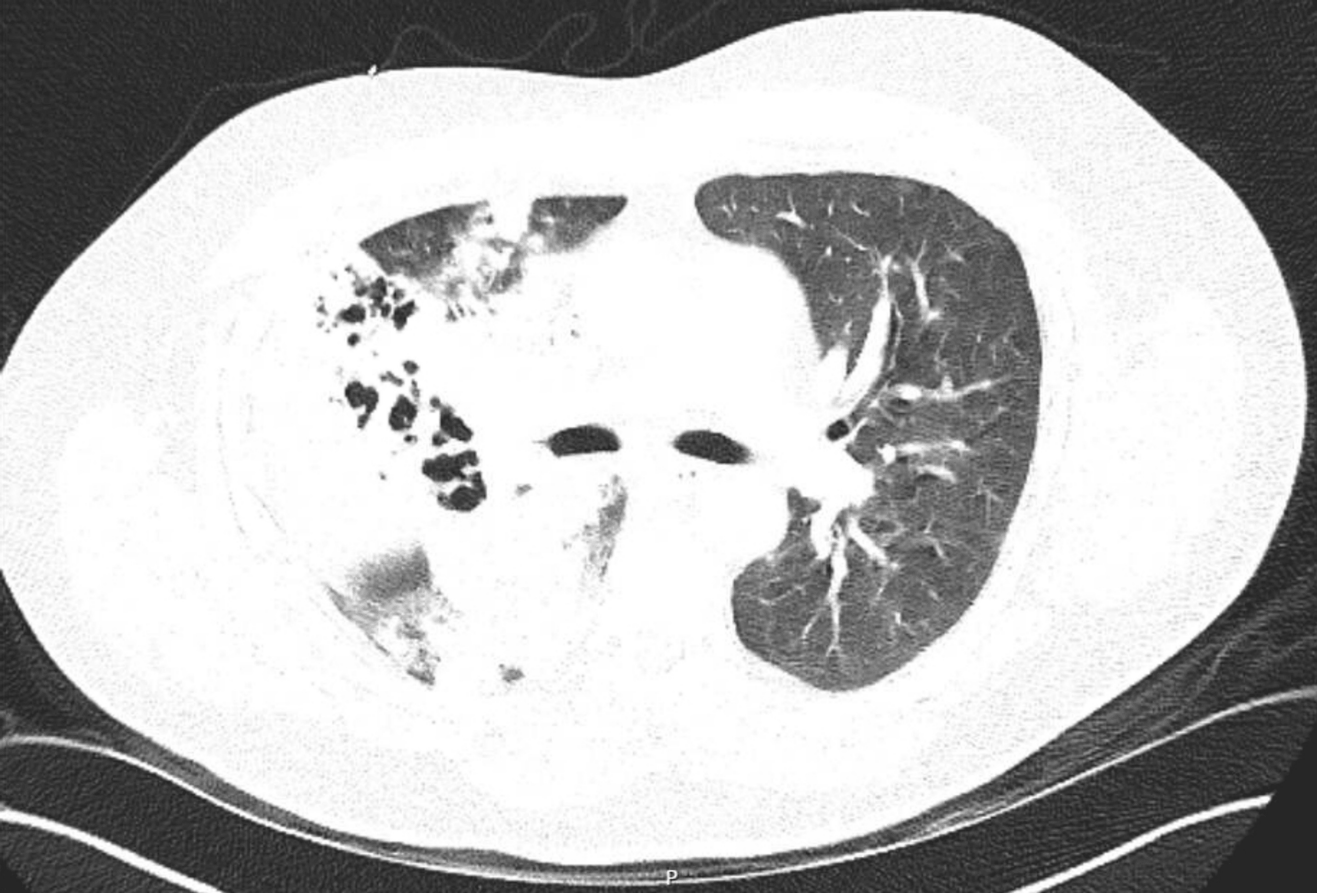
Axial CTA chest image demonstrating dense consolidation in the right upper and middle lobes of the lung. CTA, computed tomography angiography

**Figure 2:**
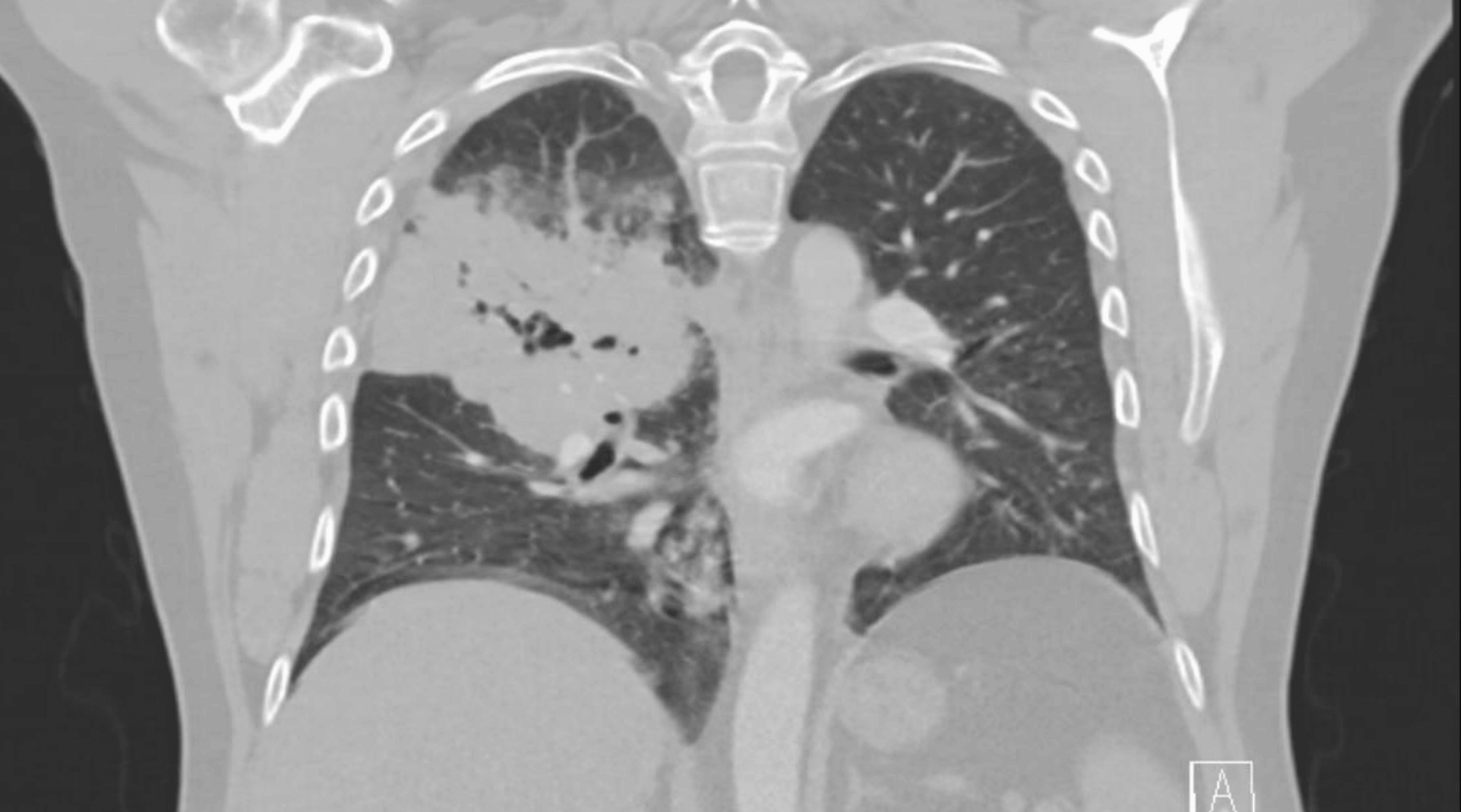
Coronal CTA chest image demonstrating dense consolidation in the right upper and middle lobes of the lung. CTA, computed tomography angiography

The patient failed outpatient treatment for presumed community-acquired pneumonia and presented to a local ED with persistent dyspnea. Chest X-ray demonstrated dense consolidation in the right upper lobe of the lung. He subsequently underwent computed tomography angiography (CTA) of the chest, which demonstrated extensive consolidative and nodular opacities in the right lung. Numerous nodular densities were present in the right upper and middle lobes of the lung. An area of consolidation measuring 8.4 × 2.7 × 3.9 cm with central cavitation was present in the right lung (Figures 1-2).

During that admission, the patient underwent flexible bronchoscopy, which was notable for endobronchial lesions that were biopsied and found to be culture-positive for blastomycosis. The patient was initiated on liposomal amphotericin B for four weeks, followed by voriconazole for one year. While on amphotericin B, the patient reported persistent headaches and underwent a lumbar puncture. Cerebrospinal fluid cultures were negative, but blastomycosis antigen testing was positive. The patient was also found to have violaceous nodules over the right leg and upper chest. Given the presentation timeline, these lesions were presumed to represent skin involvement.

**Figure 3:**
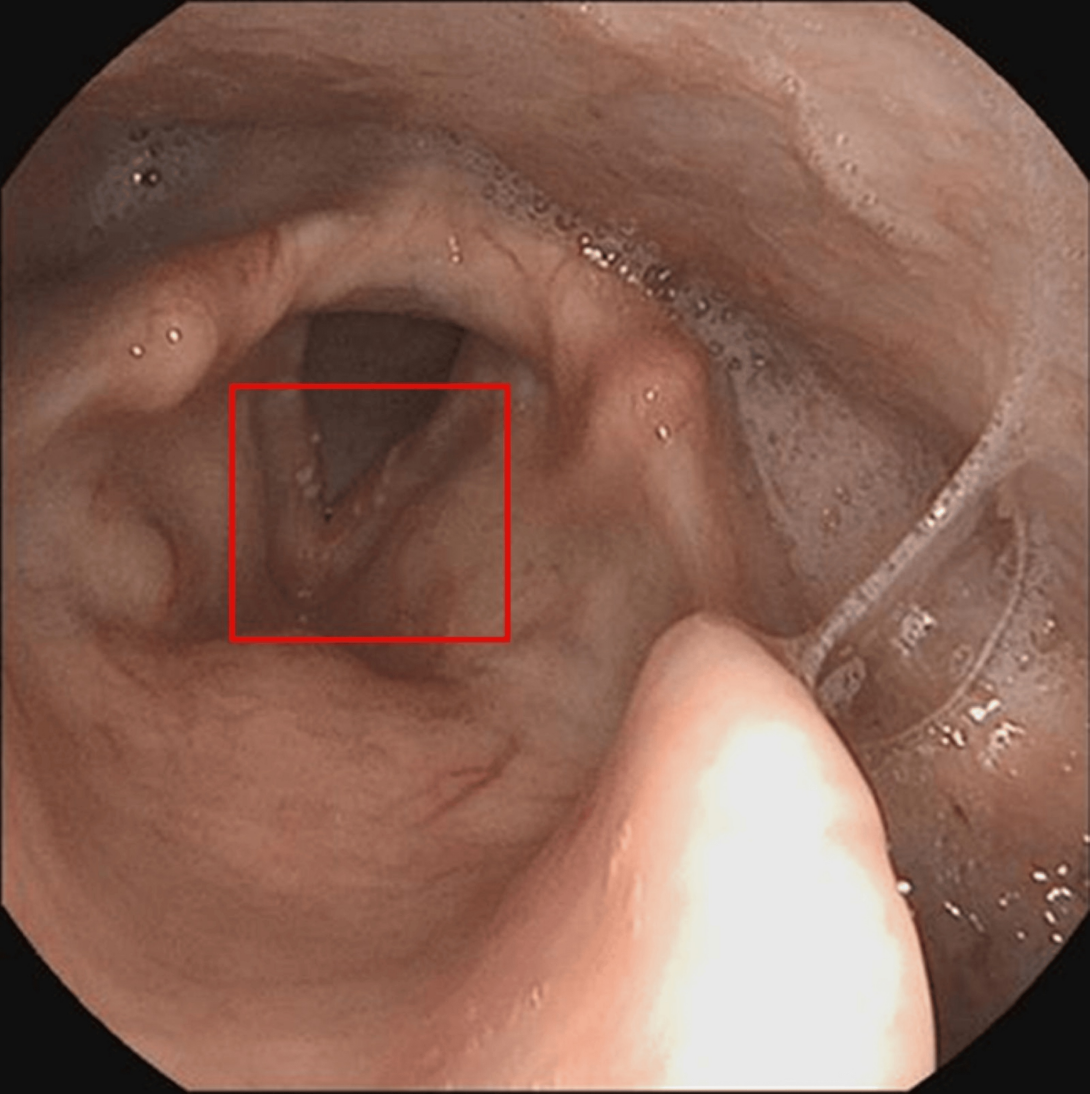
Laryngoscopy showing evidence of fungal elements on the vocal chords.

In the outpatient setting, the patient was also referred to otolaryngology for voice hoarseness. Video laryngoscopy revealed white plaques in the larynx, presumed to represent ongoing blastomycosis infection (Figure 3). After initiation of treatment, follow-up laryngoscopy one month later showed complete resolution of the fungal elements. 

Ultimately, the patient was transitioned to voriconazole after 24 days of treatment with liposomal amphotericin B due to the development of acute kidney injury. An extensive history was obtained from the patient after discharge. He worked in an office setting, making occupational exposure unlikely. However, he enjoyed outdoor activities, including bicycle riding, sometimes for many miles. Additionally, he reported going on a vacation that included a cave exploration tour. 

**Figure 4:**
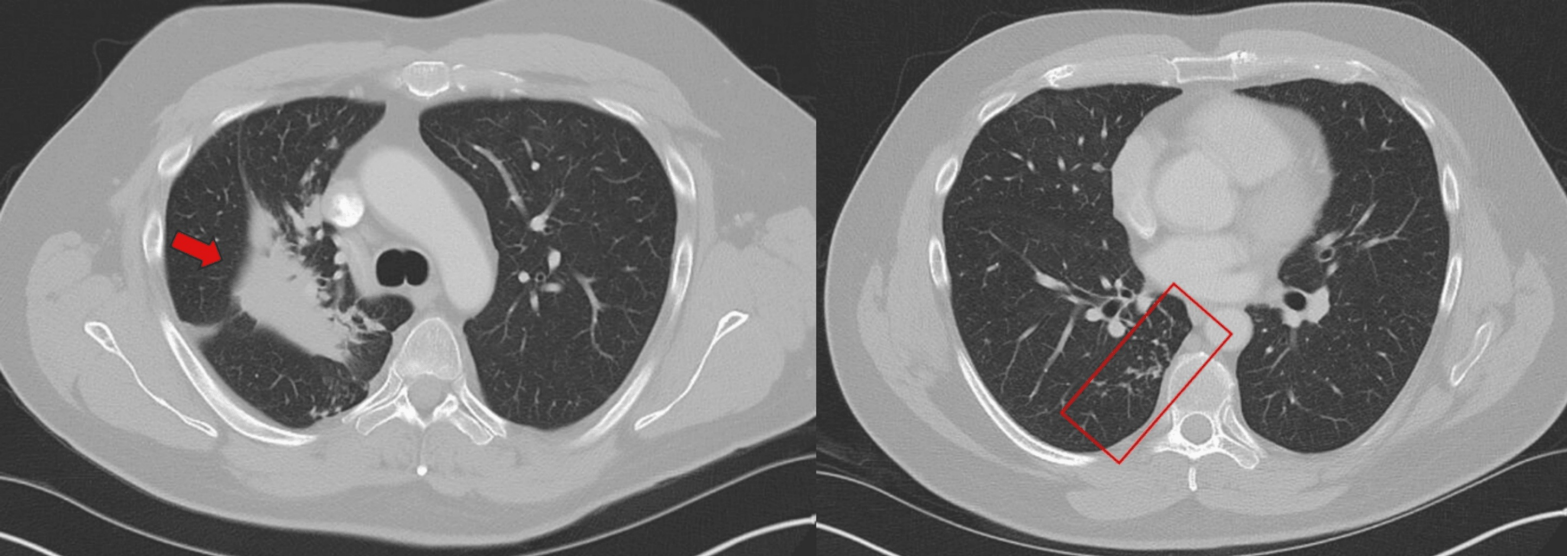
Noncontrast CT of the chest demonstrating residual fibroatelectasis (arrow) and tree-in-bud opacities (box).

After 10 months of therapy with voriconazole, the patient developed vision changes and a rash and was therefore transitioned to posaconazole due to concern for a drug-induced hypersensitivity reaction. Interval imaging with noncontrast CT of the chest, performed one year after the initial diagnosis, showed significant improvement, with residual fibroatelectatic changes in the posterior segment of the right upper lobe and minimal tree-in-bud opacities (Figure 4).

**Figure 5:**
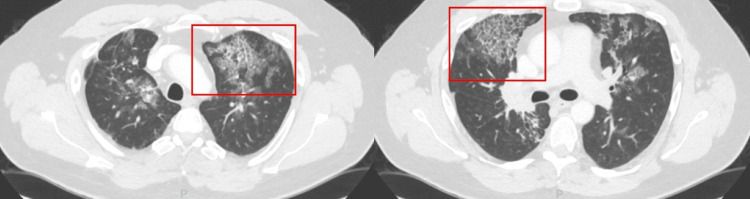
CT of the chest with intravenous (IV) contrast demonstrating bilateral areas of interlobular septal thickening.

This indicated a successful response to therapy, especially when considered alongside the clinical improvement in the patient’s dyspnea. He was continued on therapy due to persistent antigenuria. A repeat CSF analysis was performed and was negative for blastomycosis antigen. A repeat CT of the chest with intravenous (IV) contrast, obtained two years after the initial diagnosis of blastomycosis, showed a persistent right upper lobe mass with new multifocal ground-glass opacities in the bilateral upper lobes, right middle lobe, and right lower lobe, along with areas of interlobular septal thickening (Figure 5).

The patient was then referred to pulmonology for repeat bronchoscopy with bronchoalveolar lavage. No endobronchial lesions were noted, and secretions were minimal. Blastomycosis antigen, aspergillosis galactomannan, and (1,3)-beta-D-glucan testing were negative, but fungal cultures grew one colony of *Aspergillus niger* and rare *Penicillium*. Given the negative antigen testing, *A. niger* was presumed to represent colonization. A follow-up CT of the chest was obtained and showed improvement in the infiltrates, and antifungal therapy was continued.

Current admission

**Figure 6:**
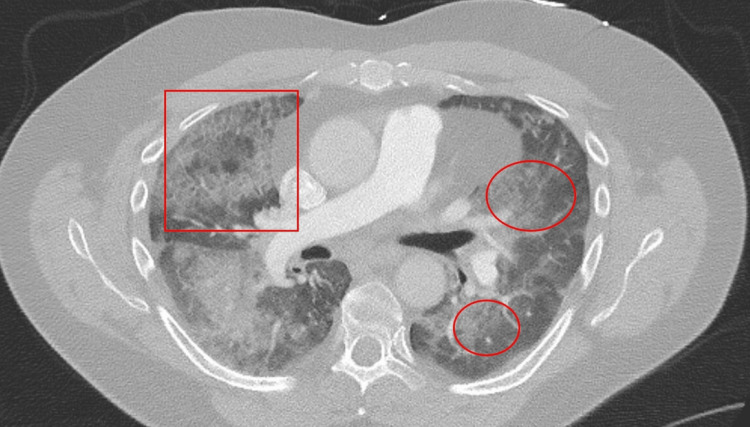
Computed tomography angiography (CTA) of the chest demonstrating a crazy-paving pattern (box) with superimposed ground-glass opacities (circles).

On presentation to the ED, the patient was afebrile and hemodynamically stable but exhibited hypoxia, with an oxygen saturation of 88% on room air during ambulation, as demonstrated by pulse oximetry. Pulmonary auscultation revealed diffuse bilateral fine crackles. Initial laboratory workup, including a complete blood count and comprehensive metabolic panel, was unremarkable. Infectious disease workup, including HIV testing, sputum culture, blood cultures, serum Mycoplasma IgG, Streptococcus pneumoniae urine antigen, and Legionella urine antigen testing, was negative. IgA, IgG, and IgM testing was also completed and was within normal limits. CTA of the chest was negative for pulmonary embolism but demonstrated extensive bilateral pulmonary opacities characterized by a *crazy-paving* pattern (septal thickening superimposed on ground-glass opacities) (Figure 6).

**Figure 7:**
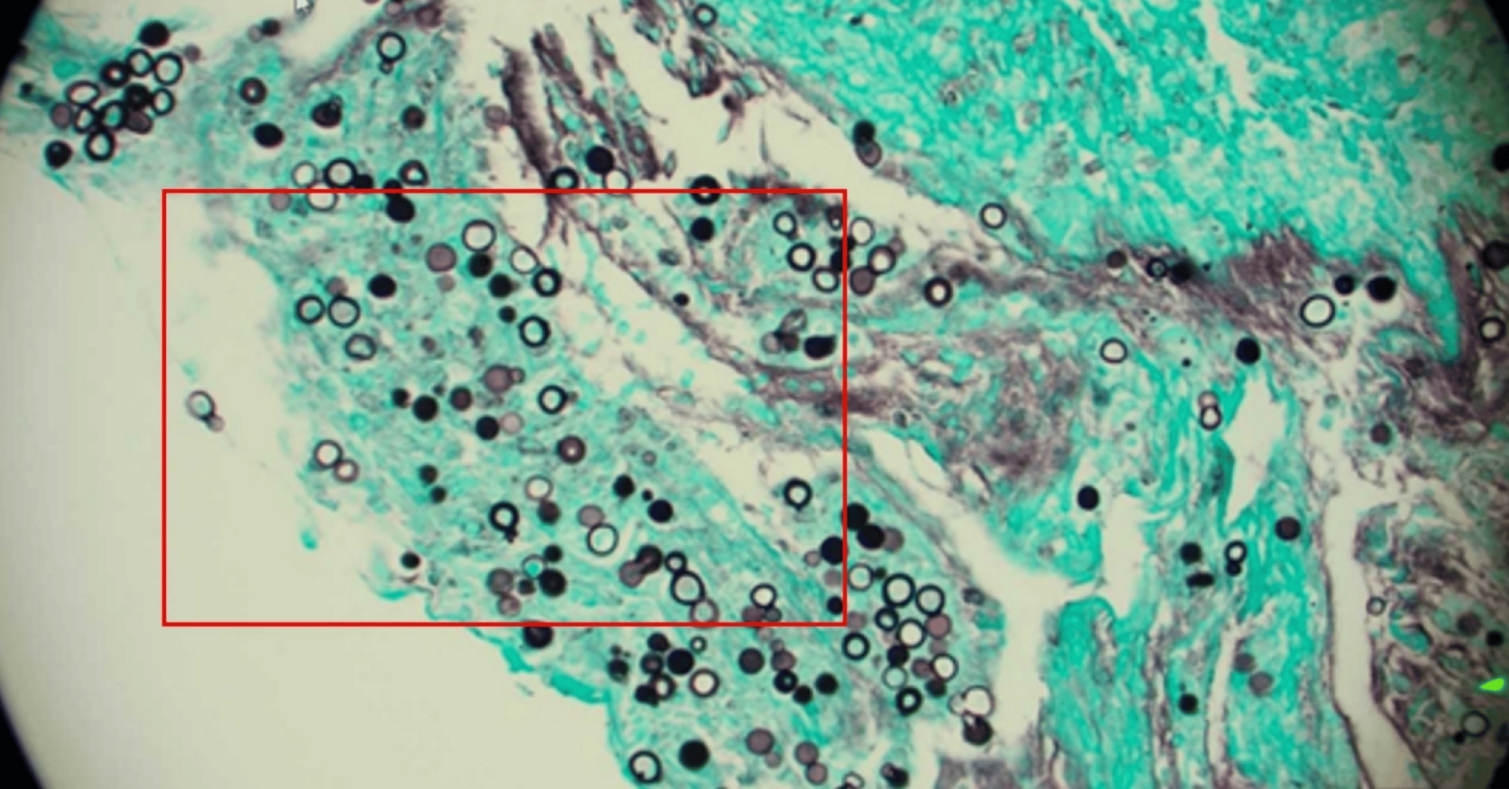
Grocott methenamine silver (GMS) stain demonstrating broad-based budding yeast forms morphologically consistent with Blastomyces (box).

**Figure 8:**
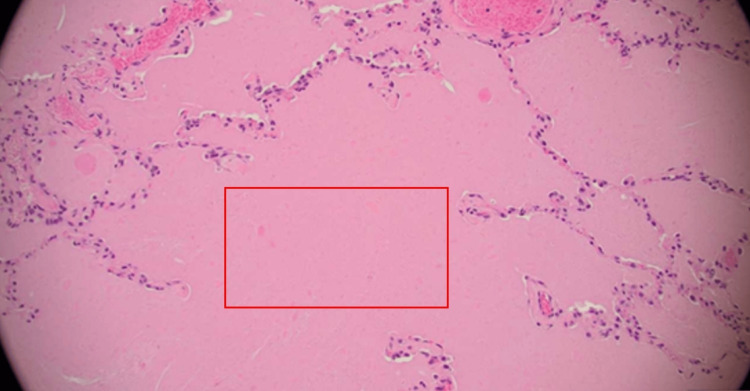
Periodic acid-Schiff (PAS) stain demonstrating alveolar spaces filled with granular eosinophilic proteinaceous material (box).

These findings had markedly progressed compared to prior imaging. The patient was admitted for hypoxic respiratory failure. Due to the progression seen on CT imaging, the differential diagnosis was broad and included worsening disseminated blastomycosis, superimposed bacterial pneumonia, early acute respiratory distress syndrome, cardiogenic pulmonary edema, diffuse alveolar hemorrhage, and a new opportunistic endemic fungal infection, among others. Given the radiographic progression despite azole therapy with therapeutic drug levels, he was reinitiated on amphotericin B. The patient was also treated empirically for bacterial pneumonia with azithromycin and piperacillin-tazobactam. Video-assisted thoracoscopic surgery (VATS) was expedited due to failed medical management, worsening CT findings, and persistent parenchymal changes despite adherence to antimicrobial therapy. The patient underwent wedge resections of the right middle and lower lobes. Histopathological examination of the lung tissue revealed areas of patchy granulomatous inflammation. Within these areas, histiocytes and multinucleated giant cells containing broad-based budding yeast forms morphologically consistent with *Blastomyces *were identified (Figure 7). Distinct from the granulomas, the alveolar spaces were filled with granular eosinophilic proteinaceous material. A periodic acid-Schiff (PAS) stain was performed and was strongly positive, confirming the diagnosis of PAP (Figure 8).

The patient was discharged five days postoperatively on 4 L of oxygen. The remainder of his hospital course was uneventful. He completed 30 days of treatment with amphotericin and was transitioned back to itraconazole 200 mg twice daily. Outpatient pulmonary function testing (PFT) showed mild to moderate restrictive lung disease with moderately to severely reduced total lung capacity and diffusion capacity. Given the histopathologic diagnosis, he was referred to another center, where further laboratory testing was completed: anti-GM-CSF level was 236 pg/mL, GM-CSF signaling was 0 units/mL blood (normal limits >216; abnormal <20), and GM-CSF inhibition was 4918.5 ng/mL blood (normal limits <20; abnormal >106). Given these laboratory results, the patient was confirmed to have autoimmune PAP. The patient subsequently underwent whole-lung lavage. After the procedure, the patient reported 70% improvement in his symptoms. The patient was also started on inhaled GM-CSF therapy. On follow-up, he was saturating well on room air at rest but continued to require 2 L of oxygen at nighttime and 5 L with activity. He continues on itraconazole and plans to pursue a second whole-lung lavage (Table 1).

**Table 1:** Outpatient pulmonary function testing. FVC, forced vital capacity; FEV1, forced expiratory volume in 1 second; TLC, total lung capacity; RV, residual volume; DLCO, diffusion capacity of the lungs for carbon monoxide; PFT, pulmonary function testing

PFT	Capacity (L)	% predicted
FVC	3.57	71
FEV1	3.11	77
FEV1/FVC ratio	-	87
TLC	4.37	57
RV	1.03	55
DLCO	-	42

## Discussion

This case illustrates a complex diagnostic dilemma: a patient with a known chronic fungal infection presenting with worsening respiratory failure despite appropriate antimicrobial therapy. The differential diagnosis in such cases typically includes refractory infection, drug toxicity, and organizing pneumonia. However, the diagnosis of PAP represents a rare intersection of infectious and autoimmune pulmonary pathology.

PAP results from the inability of alveolar macrophages to clear surfactant. In autoimmune PAP, anti-GM-CSF IgG antibodies sequester GM-CSF, preventing alveolar macrophage maturation [2]. In secondary PAP, macrophages are functionally overwhelmed or reduced in number due to conditions like myeloid leukemia or toxic inhalation [2,9]. Literature on the co-occurrence of Blastomycosis and PAP has been documented only once [8]. Secondary PAP has been described in cases of massive infection (e.g., Nocardia, Tuberculosis, or Pneumocystis) where the microorganism load overwhelms macrophage phagocytic capacity [9]. *Blastomyces*, a thermally dimorphic fungus, interacts with components of the innate immune system within the lungs. Following inhalation of the conidia, alveolar macrophages engulf the organisms, facilitating the development of the yeast form of the fungus. These yeast forms express a protein known as *Blastomyces* adhesin-1 (BAD-1) on the cell wall. This protein inhibits the production of tumor necrosis factor-alpha (TNF-alpha) by macrophages and neutrophils, suppressing the immune response and allowing survival of the yeast. In addition, BAD-1 also inhibits CD4 T-lymphocyte activation [10,11]. Thus, pulmonary infection is usually the primary site of entry for the fungus and the site from which it disseminates.

In our case, the patient’s diagnosis was confirmed through autoimmune PAP serum testing, which demonstrated positive anti-GM-CSF antibodies in conjunction with absent GM-CSF signaling and elevated inhibition levels. Given that secondary infections are common and may account for up to 20% of deaths in autoimmune PAP, prompt and aggressive treatment is warranted [12]. The patient reported hobbies involving outdoor activities, such as biking, which likely explains his exposure to blastomycosis. A few months before his initial presentation, the patient also reported going on vacation and participating in a cave exploration tour.

The radiographic finding of a *crazy-paving* pattern is highly suggestive of PAP but is nonspecific and can be seen in lipoid pneumonia, alveolar hemorrhage, and bacterial infections [13]. Therefore, histopathologic confirmation via surgical biopsy was essential. The biopsy showed the classic intra-alveolar eosinophilic material alongside broad-based budding yeast consistent with *Blastomyces *[7]. Interestingly, interlobular septal thickening was noted on CT imaging almost a year and a half before the patient’s final diagnosis. This pattern was not previously apparent, which may have been secondary to the dense infiltrates present on the initial scans.

Management of these concurrent conditions is challenging. The standard of care for symptomatic autoimmune PAP is whole-lung lavage (WLL) to mechanically remove the proteinaceous material, along with initiation of exogenous GM-CSF therapy [12]. However, GM-CSF therapy involves immune modulation, and WLL carries procedural risks, particularly in lungs with an active fungal burden. Treatment requires a delicate balance between aggressive antifungal coverage and management of the alveolar dead space caused by PAP.

## Conclusions

This case represents the second documented case of autoimmune PAP in a patient with chronic disseminated blastomycosis. It highlights the importance of broadening the differential diagnosis in patients with chronic pulmonary infections who exhibit worsening hypoxemia despite guideline-directed antifungal therapy. Early recognition through surgical biopsy was critical in this case to differentiate between fungal progression and the development of alveolar proteinosis, thereby guiding appropriate management strategies.
